# Morphology and Kinetics Evolution of Nanoscale Phase in Fe–Cr Alloys under External Strain

**DOI:** 10.3390/nano9020294

**Published:** 2019-02-19

**Authors:** Lihui Zhu, Yongsheng Li, Shujing Shi, Zhengwei Yan, Jing Chen, Shahid Maqbool

**Affiliations:** 1 School of Materials Science and Engineering, Nanjing University of Science and Technology, Nanjing 210094, China; lhzhu@njust.edu.cn (L.Z.); sjshi@njust.edu.cn (S.S.); zhengweiyan@njust.edu.cn (Z.Y.); jchen@njust.edu.cn (J.C.); shahid@njust.edu.cn (S.M.); 2 MIIT Key Laboratory of Advanced Metallic and Intermetallic Materials Technology, Nanjing 210094, China

**Keywords:** nanoscale phase, kinetics, morphology, external strain, simulation

## Abstract

Uniaxial strain was applied to aging Fe–Cr alloys to study the morphological orientation and kinetics of the nanoscale α′ phase by utilizing phase-field simulation. The effects of applied uniaxial compressive and tensile strain on the two and three-dimensional morphology as well as on the separation kinetics of the α′ phase are quantitatively clarified. Compared with the applied uniaxial tensile strain, the applied uniaxial compressive strain shows a greater effect on the rate of phase separation, lath shape morphology and an increased rate of growth and coarsening in the α′ phase, the boundary of the α + α′ phase region is widened influenced by the applied compressive strain, while the applied tensile strain results in an increase of particle number density and a decrease of particle radius. The peak value of particle size distribution of the α′ phase increases with aging time, while an opposite trend is shown under the applied strain, and there is an obvious deviation from the theoretical distribution of Lifshitz–Slyozov–Wagner under compressive strain. The orientation morphology and kinetic change show the substantial effects of applied strain on the phase separation and supplies the method for the morphological control of nanoscale particles.

## 1. Introduction

As one of the potential candidates for cladding and structural materials in future fusion reactors, Fe–Cr alloys, the basic alloy of high-chromium duplex stainless steels, exhibit a combination of beneficial properties [1,2,3,4]. The separation of the two-phase mixture of Fe-rich α phase and nanoscale Cr-rich α′ phases in ferrite [1,3] can induce embrittlement by enhancing the hardness of the alloys.

Therefore, the phase separation in Fe–Cr alloys has attracted a great deal of attention [5,6,7,8,9,10,11]. The clustering of Cr atoms inside the miscibility gap of Fe–Cr alloys was studied by Zhou et al. [1] by kinetic Monte Carlo simulations and atom probe tomography (APT) experiment. Dahlström et al. [8] studied the initial stage phase separation in Fe–Cr alloys by 3D-APT, and their results showed that phase separation is related to the nanostructure evolution and that Cr-rich regions form at the initial stages of decomposition. Li et al. [9] focused on the effects of aging temperature and applied strain on morphology by two-dimensional (2D) phase-field simulation, and showed that the orientation of the Cr-rich phase is intensified as the temperature rises in 2D simulation. Yan et al. [11] studied the evolution of the Cr-rich phase in a Fe-35 at. % Cr alloy by using 3D-APT and 3D phase-field simulation, where the 3D simulation morphology and composition show good agreement with the experimental results of APT and transmission electron microscope (TEM). Furthermore, Barker et al. [2] discussed the effect of concentration on the spinodal decomposition of Fe–Cr alloys, and concluded that the qualitative morphology is very similar between 2D and 3D simulations, while the amplitude is different. Therefore, the difference between 2D and 3D simulation results for the phase separation in Fe–Cr alloys is still not clearly clarified and needs further study.

It is found that without external strain, the separation of individual phases is affected by the elastic strain energy induced by composition inhomogeneity and the interfacial energy between precipitates and matrix phase [12,13]. The external applied strain has a substantial effect on the phase separation and spatial morphology of the second phase [14,15,16,17,18,19], which affects the mechanical, electrical and magnetic properties [20]. Essentially, the external applied strain affects the phase transformation and microstructure of the structural materials subjected to loading at high temperatures. Applied tensile or compressive stress induces the orientation morphology of precipitates [21], and directional coarsening is observed when annealed with tensile and compressive stresses [22,23,24]. The orientation of the α′ phase in Fe–Cr alloys is perpendicular to the applied tensile strain, and the early-stage phase separation is accelerated with the elevated temperature [25]. Prikhodko and Ardell [26,27,28] found that applied compressive stress retards the growth of the precipitates and widens the distribution of particle size [26]. Therefore, the effects of tensile and compressive strain on the morphology and kinetics evolution of Fe–Cr alloys are of theoretical and practical importance.

In this work, first, a comparison of 2D and 3D phase-field simulations is performed for Fe-25 at. % Cr alloy aged at 750 K, then the effects of the applied uniaxial tensile and compressive strain on the morphology, and on the kinetics of the α′ phase in Fe-28 at. % Cr alloy aged at 750 K, are quantitatively studied by 3D phase-field simulation; the rate of phase separation and later stage coarsening are clarified by the variation of volume fraction and the particle size variation.

## 2. Materials and Methods 

### 2.1. Elastic Strain Energy

The external applied strain $\varepsilon_{ij}^{a}$ works as part of the elastic strain $\varepsilon_{ij}^{\mathrm{el}}=\varepsilon_{ij}^{a}+\varepsilon_{ij}-\varepsilon_{ij}^{0}$, where a total strain of $\varepsilon_{ij}^{tot}=\varepsilon_{ij}^{a}+\varepsilon_{ij}$ is defined, $\varepsilon_{ij}$ is the internal strain and $\varepsilon_{ij}^{0}$ the eigenstrain [29,30]. The eigenstrain $\varepsilon_{ij}^{0}=\varepsilon_{0}\delta_{ij}\Delta c$ is caused by the composition heterogeneity of precipitates and the matrix, where $\varepsilon_{0}$ is the composition expansion coefficient of the lattice parameter, $\delta_{ij}$ is the Kronecker–delta function, and $\Delta c=c-c_{0}$, where *c* is the composition of Cr and $c_{0}$ is the initial average composition. The internal strain can be given by solving the mechanical equilibrium equation [30]. Then, the elastic strain energy density is expressed by

(1) $$E_{\mathrm{el}}=\frac{1}{2}C_{ijkl}(\varepsilon_{ij}^{a}+\varepsilon_{ij}-\varepsilon_{ij}^{0})(\varepsilon_{kl}^{a}+\varepsilon_{kl}-\varepsilon_{kl}^{0})$$

where *C_ijkl_* is the elastic modulus.

### 2.2. Phase-Field Model

The morphology evolution of the Cr-rich phase with the body-centered-cubic (bcc) crystal structure in Fe–Cr alloys can be described by the conservative composition field, through the Cahn–Hilliard diffusion equation [31]:

(2) $$\frac{\partial c\left(r,t\right)}{\partial t}=V_{m}^{2}\nabla \cdot \left[M\cdot \nabla \left(\frac{\delta F}{\delta c}\right)\right]$$

where *t* and $r=\left(r_{1},r_{2},r_{3}\right)$ are time and spatial coordinates, respectively. *M* is the chemical mobility [32,33]: $M=\frac{1}{V_{m}}\left[cM_{\mathrm{Fe}}+\left(1-c\right)M_{\mathrm{Cr}}\right]c\left(1-c\right)$, where *M*_Fe_ and *M*_Cr_ are atomic mobilities of Fe and Cr, respectively, and are given by the Einstein relation, $M_{i}=D_{i}/RT$, where *i* denotes the atom Fe or Cr, *R* is the gas constant, *T* is the absolute temperature, and *D_i_* is the diffusion coefficient of the *i*-th atom.

The total free energy *F* [34] of Fe–Cr alloys, containing the chemical free energy *G*, interfacial energy and elastic strain energy *E*_el_ is as follows:

(3) $$F=\int_{V}\left\{\frac{1}{V_{m}}\left[G+\frac{1}{2}\kappa {\left(\nabla c\right)}^{2}\right]+E_{\mathrm{el}}\right\}dV$$

where *V*_m_ is the molar volume of the alloy, the gradient energy coefficient *κ* is assumed as a constant for the convenience of the numerical solution of the Chan–Hilliard equation [35].

### 2.3. Numerical Calculation

By substituting Equation (3) into Equation (2), the composition evolution equation is given by

(4) $$\frac{\partial c\left(r,t\right)}{\partial t}=V_{m}\nabla \cdot \left[M\nabla \left(\frac{\delta G}{\delta c}-\kappa \nabla^{2}c+V_{m}\frac{\delta E_{\mathrm{el}}}{\delta c}\right)\right]$$

By introducing the dimensionless spatial coordinates $r^{*}=r/l$, and time $t^{*}=tD/l^{2}$, where *l* is the grid length and chosen as the average lattice constant of Fe and Cr, $D=10^{-24}$ m^2^·s^–1^ is the diffusion coefficient used for dimensionless, and Equation (4) is transformed into a dimensionless form for numerical solution:

(5) $$\frac{\partial c\left(r^{*},t^{*}\right)}{\partial t^{*}}=\nabla^{*}\cdot \left[M^{*}\nabla^{*}\left(\frac{\delta G^{*}}{\delta c^{*}}-\kappa^{*}{\left(\nabla^{*}\right)}^{2}c+\frac{\delta E_{\mathrm{el}}^{*}}{\delta c}\right)\right]$$

where $\nabla^{*}=\partial /\partial \left(r/l\right)$, $M^{*}=V_{m}RT_{c}M/D$, $G^{*}=G/RT_{c}$, $\kappa^{*}=\kappa /RT_{c}l^{2}$, $E_{\mathrm{el}}^{*}=V_{m}E_{\mathrm{el}}/RT_{c}$, and *T*_c_ = 900 K is the critical temperature of spinodal decomposition of the Fe–Cr alloy [18,36]. The dimensionless grid size is $\Delta x^{*}=\Delta y^{*}=\Delta z^{*}=1.0$ and the time step is $\Delta t^{*}=0.001$. The simulation cell is set as 128Δx*×128Δy*×128Δz* for 3D simulation, and for 2D simulation it is 512Δx*×512Δy* in order to contain enough particles for statistical accuracy. The elastic constants of the precipitates and matrix are referred to in the literature [37,38]. Also, in calculation, the magnitude of the initial thermal fluctuations [−0.003, 0.003] is added into the composition to trigger the phase separation.

## 3. Results and Discussions

### 3.1. Morphological Evolution of the α′ Phase

In this section, the morphologies of the 2D and 3D simulations were quantitatively compared under the uniaxial applied tensile strain along the x*-direction in the aging Fe–Cr alloy; the particle number of α′ phase is counted as about 200 in the 2D simulation and 300 in the 3D simulation at the initial separation stage.

**Figure 1:**
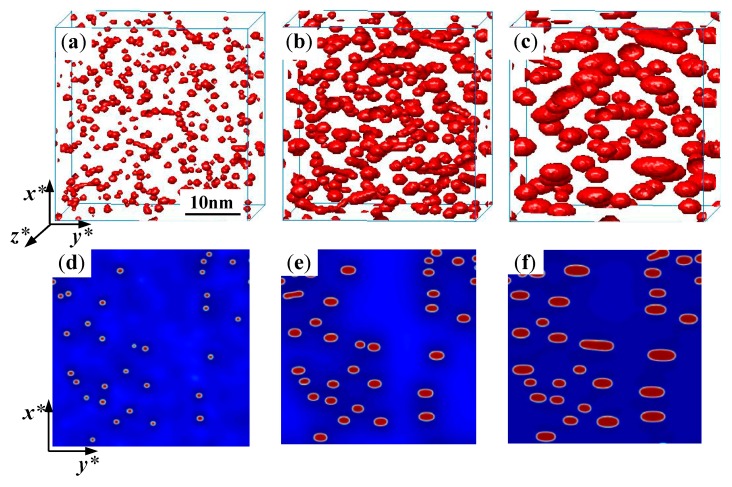
The temporal evolution of α′ phase in Fe-25 at. % Cr alloy aged at 750 K under the applied uniaxial tensile strain 0.02, (**a**,**d**) *t* = 115 h, (**b**,**e**) *t* = 805 h, (**c**,**f**) *t* = 3450 h, (**a**–**c**) 3D morphology, (**d**–**f**) 2D morphology.

Figure 1a–c shows the 3D perspective morphology of the α′ phase in Fe-25 at. % Cr alloy aged at 750 K under the applied strain $\varepsilon_{xx}^{a}=0.02$. Figure 1d–f shows the 2D morphology aged at the same time corresponding to Figure 1a–c, respectively, and the 2D morphology of the α′ phase is shown in a cell with $256\Delta x^{*}\times 256\Delta y^{*}$. It can be seen that, under the influence of the applied tensile strain with the increase in aging time, the α′ phase particles change from the initial near-spherical to an elongated shape along the y*-direction as shown in Figure 1. This orientation of the α′ phase depends on the sign of eigenstrain and the applied strain, which is illustrated in detail by Li and Chen [29]; the elongated direction can be deduced from the ratio δ of the shear modulus of the precipitates to the matrix phase [39]. Under the tensile strain, the soft α′ phase in the Fe–Cr alloy elongates along the y*-direction perpendicular to the direction of the applied strain [25].

The α′ phase in 2D is similar to the y*-x* view of the 3D morphology. Observed from the morphology of the α′ phase in 2D and 3D simulations, the particles elongate in the y*-direction normal to the direction of the applied uniaxial tensile strain (x*-direction), which is obvious from Figure 1c,f. It should be noted that an overlap of the α′ phase particles can be obviously observed in the plane view of 3D figures.

**Figure 2:**
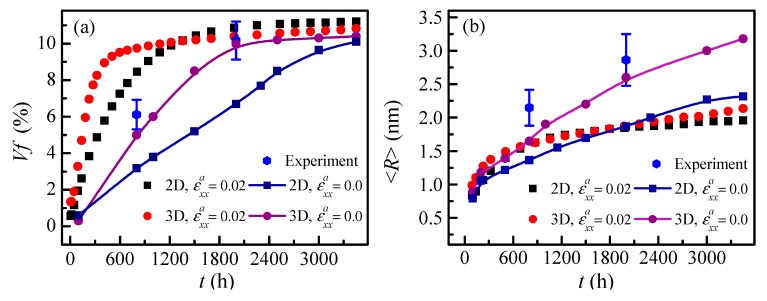
The evolution of the volume fraction *Vf* (**a**) and the average particle radius <*R*> (**b**) of α′ phase in Fe-25 at. % Cr alloy aged at 750 K under the applied uniaxial tensile strain 0.02 and 0.0 for 2D and 3D simulations, and the experimental data without applied strain.

To study the separation kinetics of the α′ phase, a comparison of the simulation results of the volume fraction and the particle radius of the α′ phase with the experimental results is performed for the state without applied strain. The experimental data of the volume fraction *Vf* and the average particle radius <*R*> of the α′ phase calculated from TEM micrographs [40] with aging for 800 h and 2000 h are depicted in Figure 2. In addition, the 2D and 3D simulation data with and without applied strain are also shown.

The simulated and experimental *Vf* and <*R*> without applied strain indicate that the α′ phase aged for 800 h is still at initial growth stage, as shown in Figure 2a,b, while the *Vf* with applied strain almost reaches the equilibrium value at 800 h, as is seen in Figure 2a. Thus, the applied strain accelerates the initial phase separation. It can also be observed that the *Vf* and <*R*> calculated from the TEM micrographs are closer to the results of the 3D simulation without applied strain, as shown in Figure 2. Moreover, the 2D simulation does not give the complete spatial structure, such as the interconnection of particles; therefore, the 3D simulation is closer to the experimental results.

It can be seen from the variation of *Vf* in Figure 2a that the phase separation shown by 3D simulation is faster than that of the 2D results. The <*R*> of the α′ phase under applied strain is smaller than that without applied strain at the coarsening stage, as shown in Figure 2b. Also, the change of <*R*> indicates that the coarsening of the α′ phase is faster in 3D simulation than that of 2D simulation; the reason for this can be attributed to the faster interconnection of neighbouring particles in 3D space and the larger driving force available for coarsening in reducing the interfacial curvature [41]. Therefore, the phase separation and coarsening rate simulated by the 3D model are faster than that of the 2D simulation.

### 3.2. Effect of Applied Strain on Separation Kinetics

In Section 3.1, as the results of 3D simulation are closer to the experimental values compared to the 2D results, the 3D simulation is therefore performed to study the kinetic evolution of the α′ phase under applied tensile and compressive strain in Fe-28 at. % Cr alloy aged at 750 K.

#### 3.2.1. Composition Boundary Change under Applied Strain

Andersson and Sundman [42] compared the spinodal line predicted by the thermodynamic model with the experimental data and showed that there is no clear boundary between the nucleation-growth and spinodal decomposition region, while a transition region exists from the nucleation growth to spinodal decomposition near the spinodal line [43], Xiong et al. [36] summarized the results for the nucleation growth and spinodal decomposition in the phase diagram; the data show an overlap around the spinodal line. To estimate the phase boundary of the α and α′ phase, we calculated the free energy including chemical and elastic energy in the Fe–Cr alloy with Cr concentration ranges from about 0.0 to 1.0 at 750 K. The equilibrium composition of phase separation is derived from the tangent of free energy curves; the boundaries of the phase separation at 750 K are about *c*_Cr_ = 17 at. %, and the critical compositions of spinodal decomposition are about *c*_Cr_ = 30 at. %. Theoretically, the phase separation happens via nucleation and growth in the metastable region with *c*_Cr_ = 17 at. % to 30 at. % at 750 K, while the spinodal decomposition happens when the composition is greater than *c*_Cr_ = 30 at. % inside the spinodal regions. The composition of *c*_Cr_ = 28 at. % is near the spinodal boundary in the metastable regions; this section is focused on the effects of tensile and compressive strain on the phase separation of α′ phase in Fe-28 at. % Cr alloy aged at 750 K. 

**Figure 3:**
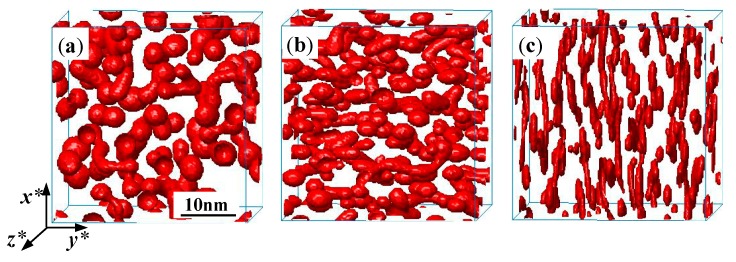
The morphology of α′ phase in the Fe-28 at. % Cr alloy aged at 750 K for *t* = 1150 h. (**a**) without applied strain $\varepsilon_{xx}^{a}=0.0$, (**b**) tensile strain $\varepsilon_{xx}^{a}=0.02$, (**c**) compressive strain $\varepsilon_{xx}^{a}=-0.02$.

An anisotropic and soft precipitate in the presence of an externally applied stress tends to become thicker and will require more energy than one which inclines to become thinner [21], such as the plate-shaped particles under compressive strain shown in Figure 3. Therefore, the separation of the α′ phase under compressive strain is easier than that of tensile strain. The Cr concentrations of the α and α′ phase in Fe-28 at. % Cr alloy aged at 750 K are calculated with applied tensile and compressive strain, as shown in Table 1. It can be seen that the boundary of Cr concentrations in the α and α′ phase under compressive strain is wider than that of tensile strain, which is consistent with the effect of compressive and tensile strain on the free energy that decides the equilibrium concentrations.

#### 3.2.2. Orientation Morphology of the α′ Phase under Applied Strain

The directional coarsening of precipitates under the applied stress will affect the mechanical properties, which is known as rafting in superalloys. Wang et al. [44] and Schmidt et al. [45] both simulated the directional coarsening of Ni-based superalloys by the phase-field model, and the rafting of the γ′ phase is observed when the external strain is tensile or compressive. In this work, the morphology of the α′ phase in Fe-28 at. % Cr alloy aged at 750 K is studied under applied uniaxial strain along the x*-direction, as shown in Figure 3, where three different strains, $\varepsilon_{xx}^{a}=0.0$, 0.02 and −0.02, are applied and are shown in Figure 3a–c, respectively.

The α′ phase particles, as shown in Figure 3a, are randomly distributed with a near-spherical shape without applied strain, while the orientation of the α′ phase is obvious when the uniaxial strain 0.02 is applied in Figure 3b. Under the tensile strain, the α′ phase is elongated in the y*-direction that is normal to the direction of the applied uniaxial strain, as illustrated in Figure 3b. However, the orientation of the α′ phase under the compressive strain is parallel to the direction of applied uniaxial strain (x*-direction) and shows a lathy shape, as depicted in Figure 3c.

Wang et al. [44] clarified that the difference of the elongated direction between the tensile and compressive strain depends on whether the phase is hard or soft, and the lattice mismatch of the precipitates is either positive or negative. Zhu et al. have demonstrated that the α′ phase is soft in Fe–Cr alloys [25]. In addition, the interactions between the applied strain and eigenstrain can cause the orientation difference of morphology for tensile or compressive strain. As shown in Fratzl’s results [21], for the soft precipitates, the matrix will prevent them from contracting along the plate, which can only contract in the direction perpendicular to the plate; hence, the anisotropic part of the strain is a contraction in this direction, and so the plate-like precipitates will be energetically favoured when the stress tends to compress them.

In the Fe–Cr alloys, a negative elastic anisotropy ($C_{11}^{M}-C_{12}^{M}-2C_{44}^{M}<0$) [46] of the matrix and a positive elastic anisotropy of precipitates coexist, and the eigenstrain of the two-phase system is a dilatational strain for a positive lattice misfit of 0.0056. When the tensile strain is along the x*-direction, the precipitates elongate in the y*-direction, while the compressive strain produces an inverse result [44]. Therefore, the orientation of the α′ phase under compressive strain is along the direction of applied strain, while it is normal to the direction of tensile strain (Figure 3b,c). The orientation of the lathy shape α′ phase also indicates the potential morphology design via the application of external strain during aging.

#### 3.2.3. Separation Kinetics of the α′ Phase under Applied Strain

**Figure 4:**
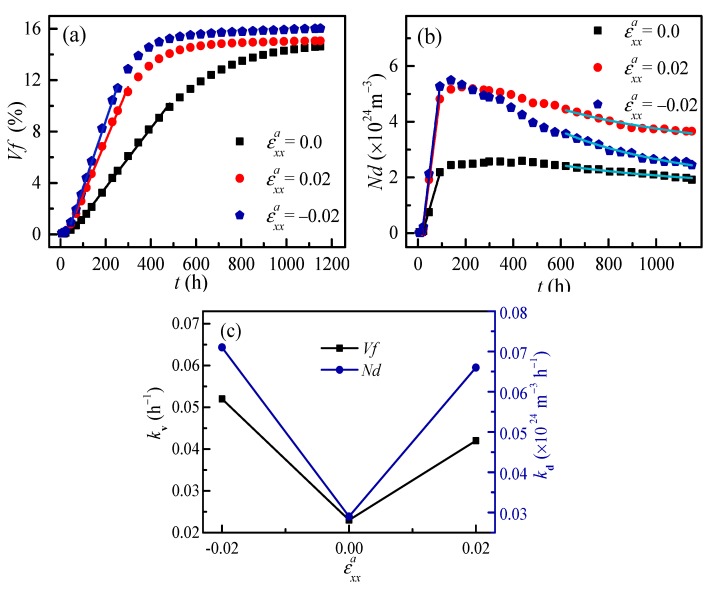
The temporal evolution of the volume fraction *Vf* (**a**) and particle number density *Nd* (**b**) of the α′ phase in Fe-28 at. % Cr alloy aged at 750 K under applied strain for 3D simulation, and the variation rates of *Vf* and *Nd* of the α′ phase as a function of applied strain (**c**).

To compare the effect of tensile and compressive strain on the kinetic evolution of the α′ phase, the same thermal fluctuation is added into the initial composition to trigger the phase separation with applied tensile and compressive strain. Figure 4a,b shows the temporal evolution of the volume fraction *Vf* and the particle number density *Nd* of the α′ phase in Fe-28 at. % Cr alloy aged at 750 K by 3D simulation. It can be seen that the values of *Vf* and *Nd* under compressive and tensile strain are greater than that without applied strain at the early stage, and the effect of compressive strain is more obvious. To reveal the kinetic change of the α′ phase under the effect of applied tensile and compressive strain, the early-stage change rates *k*_v_ of *Vf* and *k*_d_ of *Nd* of the α′ phase as a function of applied strain are shown in Figure 4c. The change rates of *Vf* and *Nd* also show a greater effect for compressive strain on the phase separation than that of tensile strain.

The equilibrium *Vf* under compressive strain is 16%, which is a little higher than the 15% for tensile strain or without applied strain. The steady-state coarsening is regarded as beginning when the equilibrium *Vf* is reached. With this definition, the steady-state coarsening begins at about 500 h for both compressive and tensile strain, while it is about 1000 h without applied strain, as shown by the *Vf* in Figure 4a. To compare the effects of tensile and compressive strain on the coarsening, the time exponent *n* for average particle radius <*R*>, and the time exponent *m* for particle number density *Nd* of α′ phase after aging for 600 h are listed in Table 2. In Table 2, the time exponent *m* of particle number density *Nd* is −0.62 under compressive strain, which is greater than the value of −0.34 under tensile strain and −0.32 without applied strain at the coarsening stage. The reason for this is that the directional coarsening (Figure 3c) leads to a fast decrease of the particle number under the compressive strain. Therefore, the compressive strain has more obvious effects on accelerating the coarsening than that of tensile strain, and the time exponents of <*R*> under compressive and tensile strain are both less than without applied strain.

**Figure 5:**
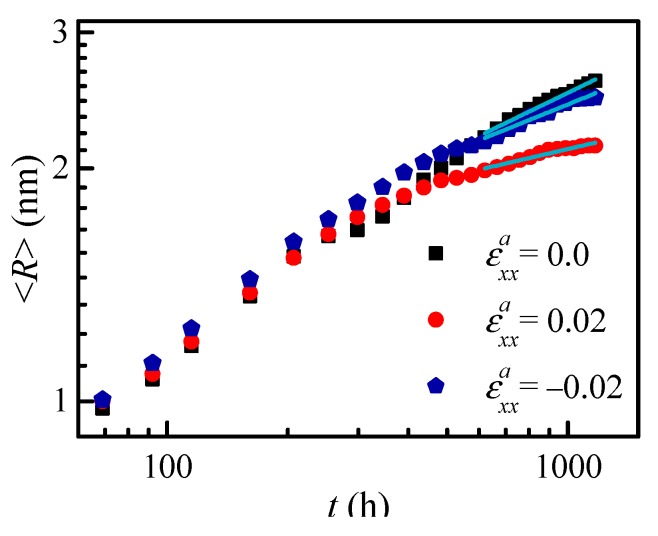
The temporal evolution of the average particle radius <*R*> of the α′ phase in Fe-28 at. % Cr alloy aged at 750 K under applied strain.

As shown in Figure 5, there is an apparent increase in the average particle radius <*R*> as the aging time increases, and the particle radius without applied strain is a little larger than that under applied strain at the steady-state coarsening stage (*t* > 600 h). The coarsening rate constant *k*_c_ is calculated to compare the effects of uniaxial tensile and compressive strain; the coarsening rate constants under uniaxial compressive and tensile strain are, respectively, *k*_c3_ = 0.01 nm^3^ h^−1^ and *k*_c2_ = 0.004 nm^3^ h^−1^, which is less than the value *k*_c1_ = 0.013 nm^3^ h^−1^ without applied strain. Kim and Voorhees [47] calculated the coarsening rate constant of Ostwald ripening under a constant equivalent radius of particles; the results show that the coarsening rate constant decreases with an increasing aspect ratio of particles. In this study, the coarsening rate constant is calculated with both coalescence coarsening and Ostwald ripening. The coarsening rate constant *k*_c2_ with high aspect ratio of 4.5 under compressive strain is greater than *k*_c1_ with an aspect ratio of 2.01 under tensile strain, which is different from Kim’s results. The difference is due to the assumption of the constant radius and increased number density by Kim et al. [47], while the particle number density decreases in the present results. 

**Figure 6:**
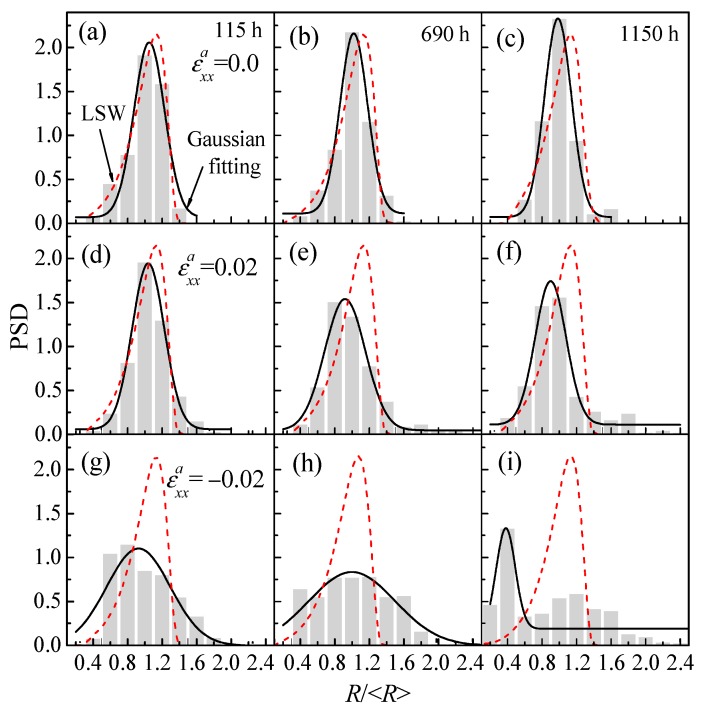
The histogram of the particle size distribution (PSD) of the α′ phase in Fe-28 at.% Cr alloy as a function of aging time aged at 750 K, (**a**–**c**) $\varepsilon_{xx}^{a}=0.0$, (**d**–**f**) $\varepsilon_{xx}^{a}=0.02$, (**g**–**i**) $\varepsilon_{xx}^{a}=-0.02$. (**a**,**d**,**g**) 115 h, (**b**,**e**,**h**) 690 h, (**c**,**f**,**i**) 1150 h.

Figure 6 shows the variation of particle size distribution (PSD) of the α′ phase with the aging time under compressive and tensile strain; the histogram is the simulation result and its Gaussian fitting is shown by the solid line, and the red dashed line is the theoretical value of Lifshitz–Slyozov–Wagner (LSW) [48]. When there is no applied strain, the peak value of the PSD rises as the aging time increases, with the peak position located at 1.0 and similar to the theoretical value of LSW, as shown in Figure 6a–c. However, the peaks start decreasing under the applied strain as aging time increases, which is lower than LSW’s prediction, as shown in Figure 6d–i. The change of the PSD peaks is caused by the additional strain energy, which changes the particle shape and spatial distribution strongly; thus, the state of the system is different to LSW’s assumptions of a dilute solution without elastic interactions.

Under the tensile and compressive strain, the width of PSD becomes broader, as shown in Figure 6d–i. The PSD indicates that the particle size is similar at the initial phase separation stage, then the α′ phases coalesce into large particles or some small particles are dissolved via Ostwald ripening, and so the width of the PSD becomes broader. It is noted that the PSD has obvious changes under compressive strain, as shown in Figure 6g–i. Due to the formation of lathy particles via the directional coarsening and arrangement along the z*-axis, the PSD deviates from the normalized distribution. The coarsening of the α′ phase results in the increase of the average particle radius <*R*> and the movement of the peak position of PSD to the left, especially for the applied compressive strain $\varepsilon_{xx}^{a}=-0.02$. In addition, the height of the histogram shows a more obvious decrease under $\varepsilon_{xx}^{a}=-0.02$ than that of $\varepsilon_{xx}^{a}=0.02$, as shown in Figure 6g–i and Figure 6d–f. Therefore, the peak value of PSD increases with the aging time without applied strain, while it decreases under the effects of applied strain, especially for the applied compressive strain.

## 4. Conclusions

The temporal morphology and the kinetics of the nanoscale α′ phase under the external applied strain in an aged Fe-25 at. % Cr alloy are investigated by two and three-dimensional phase-field simulations. The study shows that the 3D simulation is closer to the experimental results. Also, the applied uniaxial compressive strain illustrates more obvious effects on the morphological orientation and separation rate of the α′ phase than the uniaxial tensile strain, the lathy α′ phase with directional coarsening is observed, and the particle size distribution deviates from a normal distribution. In addition, the composition boundary of the α + α′ phase region can be seen to be widely influenced by the applied compressive strain.

The applied strain accelerates the initial phase separation, while the coarsening rate decreases under the applied strain for the steady-state coarsening of the α′ phase, and the applied compressive strain induces a greater change rate of growth and coarsening than that of tensile strain. The α′ phase is elongated in the direction normal to the direction of the applied uniaxial tensile strain, while the elongation of the α′ phase is parallel to the direction of uniaxial compressive strain. The peak value of particle size distribution decreases under the applied strain, especially for the compressive strain. The results are helpful for understanding the evolution of nanoscale particles under applied strain, and indicate a microstructure design with orientation arrangement.

## Figures and Tables

**Table 1:** The Cr concentration (at. %) in α and α′ phase of Fe-28 at. % Cr alloy aged at 750 K with and without applied strain.

Strain	α (at. % Cr)	α′ (at. % Cr)
$\varepsilon_{xx}^{a}=-0.02$	16.9	84.2
$\varepsilon_{xx}^{a}=0.02$	17.5	82.3
$\varepsilon_{xx}^{a}=0.0$	18.1	82.0

**Table 2:** The time exponent *m* for *Nd* and *n* for <*R*> of α′ phase in Fe-28 at. % Cr alloy aged at 750 K.

Time Exponent	$\varepsilon_{xx}^{a}=-0.02$	$\varepsilon_{xx}^{a}=0.0$	$\varepsilon_{xx}^{a}=0.02$
*m* (*Nd* ~ *t^m^*)	−0.62	−0.32	−0.34
*n* (<*R*> ~ *t^n^*)	0.21	0.25	0.12
